# Gene–diet interaction analysis using novel weighted food scores discovers the adipocytokine signaling pathway associated with the development of type 2 diabetes

**DOI:** 10.3389/fendo.2023.1165744

**Published:** 2023-08-23

**Authors:** Catherine Apio, Wonil Chung, Min Kyong Moon, Oran Kwon, Taesung Park

**Affiliations:** ^1^ Interdisciplinary Program in Bioinformatics, Seoul National University, Seoul, Republic of Korea; ^2^ Department of Statistics and Actuarial Science, Soongsil University, Seoul, Republic of Korea; ^3^ Department of Internal Medicine, College of Medicine, Seoul National University, Seoul, Republic of Korea; ^4^ Department of Nutritional Science and Food Management, Ewha Womans University, Seoul, Republic of Korea; ^5^ Department of Statistics, Seoul National University, Seoul, Republic of Korea

**Keywords:** type 2 diabetes, recommended food score, polygenic risk scores, case-control study, dietary patterns

## Abstract

**Introduction:**

The influence of dietary patterns measured using Recommended Food Score (RFS) with foods with high amounts of antioxidant nutrients for Type 2 diabetes (T2D) was analyzed. Our analysis aims to find associations between dietary patterns and T2D and conduct a gene-diet interaction analysis related to T2D.

**Methods:**

Data analyzed in the current study were obtained from the Korean Genome and Epidemiology Study Cohort. The dietary patterns of 46 food items were assessed using a validated food frequency questionnaire. To maximize the predictive power of the RFS, we propose two weighted food scores, namely HisCoM-RFS calculated using the novel Hierarchical Structural Component model (HisCoM) and PLSDA-RFS calculated using Partial Least Squares-Discriminant Analysis (PLS-DA) method.

**Results:**

Both RFS (OR: 1.11; 95% CI: 1.03- 1.20; P = 0.009) and PLSDA-RFS (OR: 1.10; 95% CI: 1.02-1.19, P = 0.011) were positively associated with T2D. Mapping of SNPs (P < 0.05) from the interaction analysis between SNPs and the food scores to genes and pathways yielded some 12 genes (CACNA2D3, RELN, DOCK2, SLIT3, CTNNA2, etc.) and pathways associated with T2D. The strongest association was observed with the adipocytokine signalling pathway, highlighting 32 genes (STAT3, MAPK10, MAPK8, IRS1, AKT1-3, ADIPOR2, etc.) most likely associated with T2D. Finally, the group of the subjects in low, intermediate and high using both the food scores and a polygenic risk score found an association between diet quality groups with issues at high genetic risk of T2D.

**Conclusion:**

A dietary pattern of poor amounts of antioxidant nutrients is associated with the risk of T2D, and diet affects pathway mechanisms involved in developing T2D.

## 1 Introduction

Diabetes is one of the most significant global public health concerns, imposing a heavy global burden on public health and socioeconomic development. Although incidence has started to decrease in some countries, the prevalence of diabetes has increased in recent decades in other developed and developing countries (1). Type 2 diabetes (T2D) makes up around 90% of cases of diabetes (2), and according to the World Health Organization, the number of people diagnosed with T2D is on the rise annually, even among young people (2).

The development of T2D is caused mainly by an interplay of unhealthy lifestyles and environmental and genetic factors. While some of these factors are under individual control, such as lifestyle, others are not, such as increasing age, sex, and genetics. Diet has also been firmly attributed to the risk of T2D (3, 4). This association has been confirmed in many prospective studies (5–8). In addition, T2D is an increasingly prevalent metabolic disorder causing severe micro- and macrovascular complications, namely, cardiovascular disease (CVD), retinopathy, neuropathy, and nephropathy (3, 9). Moreover, the beneficial effects of weight loss or lifestyle modification have been reported to prevent, delay, and reduce disease incidence (2, 10).

Therefore, valid estimation of overall dietary patterns (habitual food and nutrient intakes) has become a fundamental aspect of studying the relationships between diet and health status (8). General dietary habits can provide insights beyond the role of nutrients and single foods (2, 11). Some of the indices are based on national nutrition recommendations and national dietary guidelines that assess overall nutritional patterns, including the healthy eating index, alternate healthy eating index, healthy diet indicator, Recommended Food Score (RFS), diet quality index, Diet Quality Score, Mediterranean Diet Score (MDS), and Alternate Mediterranean Diet Score (aMDS). The RFS, MDS, and aMDS based on foods and food groups are relatively more straightforward in assessing overall dietary patterns and are based on food groups and nutrients (9, 11).

Recently, the pathophysiological influence of gene–lifestyle or gene–environment (G × E) interactions on the risk of T2D is currently under intensive research. Evidence of G × E interactions on the risk of development of T2D has been reported in many prospective studies reviewed here (3, 4). Here, G × E interaction analyses focusing on gene–diet interaction using RFS and SNPs while controlling for other confounding lifestyle factors like smoking, alcohol and coffee consumption, income and education levels, and so forth, were carried out for the Korean adult population. Odds ratios (ORs) with 95% confidence intervals (CIs) for the association and interaction analyses were calculated. Furthermore, the subjects were grouped into low, intermediate, and high diet quality groups using the food scores and genetic risk groups using an estimated global polygenic risk score (PRS), and interaction analyses between the groups were performed. Data from the Korean Genome and Epidemiology Study (KoGES) consortium, a prospective cohort study conducted in Korea in 2021, was used for our analysis (12–14).

However, a previous study using RFS for the Korean population could not show an acceptable association with the risk of T2D (11, 15, 16). This may be because the contributing power of each food item is different from each other: some food items contribute more than others. Therefore, weighted food scores were developed to maximize the unweighted RFS’s interaction and predictive power. One score, HisCoM-RFS, was proposed using a novel statistical model called the Hierarchical Structural Component model (HisCoM). HisCoM estimates the weights for each food item used in the RFS calculation. HisCoM-RFS was contrasted for comparable results in different association analyses with PLSDA-RFS, another weighted food score calculated using the known partial least squares-discriminant analysis (PLS-DA) method. It finds another set of weights for each food item without considering food group categories. Both approaches assume a linear relationship exists between food items and the outcome T2D.

## 2 Materials and methods

### 2.1 Study population

The study participants were recruited through the Korean Genome and Epidemiology Study (KoGES), a consortium established for the identification of gene–environment factors and their interactions in commonly known diseases, such as T2D, hypertension, metabolic syndrome, obesity, and cardiovascular disease in Koreans (12). KoGES is a project comprising six prospective cohort studies categorized into population-based and gene–environment model studies extensively explained elsewhere (13, 14). We focused on the KoGES Ansan–Ansung study cohort whose data collection was initiated in 2001–2002 (baseline), with follow-up examinations conducted every 2 years. The participants were unrelated Korean individuals (N = 10,038) aged 40–69 years, representing urban (Ansan) and countryside (Ansung) populations. Our analyses involved data from the baseline recruit (17). Among the KoGES cohorts, the KoGES Ansan–Ansung cohort was chosen because it possesses the Frequency Food Questionnaire and has a more extended follow-up period than other cohorts.

### 2.2 Genotype data

The genotype data of the above participants were obtained through the Korea Association Resource (KARE) project, which was established in 2007 to conduct a large-scale genome-wide association study (GWAS) of the participants recruited through the KoGES Ansan–Ansung cohorts (18). The participants’ common standard variant genotype data were generated using the Affymetrix Genome-Wide Human SNP array 5.0. The chip comprised around 50 million autosomal single-nucleotide polymorphisms (SNPs). There were 352,228 SNPs in 8,840 individuals left after quality control (QC) analysis. SNPs having minor allele frequencies<0.05, genotype calling rates<95%, and Hardy–Weinberg equilibrium P-values<10^−6^ were removed. Only participants with consistent sex and calling rates (>90%) were preserved. Missing values of existing variants were imputed after QC, and PLINK (v1.90) (19) was used during QC. The SNPs were mapped to the UCSC hg19 genomic coordination. Missing genotype data were imputed using the Beagle 5.0 (20) software program.

### 2.3 Diagnosis of T2D subjects

After participants had fasted for at least 8 h, fasting plasma glucose (FPG; mg/dL), fasting plasma insulin (FPI; IU/mL), and triglycerides (TG; mg/dL) were measured. High-performance liquid chromatography was used to measure glycosylated hemoglobin (HbA1c). The following criteria were used to determine T2D subjects: (1) taking medication any for T2D; (2) fasting plasma glucose (FPG) ≥126 mg/dL, 2-h postprandial blood glucose (Glu120) ≥200 mg/dL, or glycated hemoglobin (HbA1c) ≥6.5%; and (3) age of disease onset ≥40 years. The following criteria selected normal subjects: (1) FPG<100 mg/dL, Glu120<140 mg/dL, and HbA1c<5.7% and (2) no history of diabetes (never been diagnosed with T2D) (21, 22). If a subject does not meet these criteria, then the subject is excluded from being a normal subject.

### 2.4 Covariates

We selected 10 covariates as adjustment and lifestyle factors for control during the analysis. This included age, sex, area (urban or village), body mass index (BMI), smoking, alcohol consumption, coffee consumption, metabolite equivalents (physical activity), education level, and income level. The covariates were assessed using self-administered questionnaires. The monthly household income is categorized into eight groups (0.5, 0.5~1, 1~1.5, 1.5~2, 2~3, 3~4, 4~6, and >6 million Korean won). Here 1,000 Korean won approximately corresponded to 0.9 US dollars. Smoking was categorized into non-smokers as well as former, occasional, and habitual smokers. Alcohol consumption was categorized into non-drinkers, former drinkers, and current drinkers. Time spent during five physical activity states (inactive, very light, light, moderate, and intense) were classified into nine ranges (0; none, 1;<30 min, 2; 30~60 min, 3; 60~90 min, 4; 90~2 h, 5; 2~3 h, 6; 3~4 h, 7; 4~5 h, 8; >5 h). These were converted to metabolic equivalents (METs) according to (17) (1.0 for inactive, 1.5 for very light, 2.4 for light, 5.0 for moderate, and 7.5 for intensive). The BMI (kg/m^2^) of the participants was computed by dividing the weight (nearest 0.1 kg) in kilograms by the height (measured to the nearest 0.1 cm) in square meters. A further detailed description of the characteristics of the KoGES cohort can be found here (23). The list of the covariates used in our analyses is shown in **Supplementary Table 1**.

### 2.5 Dietary assessment

Dietary assessment was done through a validated semiquantitative food frequency questionnaire (FFQ) (24, 25), which records the consumption frequencies and portion sizes of 106 (Ansan and Ansung study) food items and drinks consumed during the previous year. The FFQ consisted of nine categories: never or seldom, once a month, one to two times a week, two to three times a week, three to four times a week, five to six times a week, once daily, twice daily, or more than three times daily. Furthermore, their daily frequency of meals was recorded as one meal a day, two meals a day, three meals a day, more than four meals a day, or irregular.

### 2.6 Recommended food score

Intake information from the FFQ was used to calculate the study subjects’ RFS. RFS measures the overall dietary pattern of the individuals, a food tally based on reported consumption of foods bearing high amounts of antioxidant nutrients, consistent with the current American dietary guidelines of Kant et al., modified for the Korean population (11, 15, 16). A total of 45 food items (10 food groups) and one response for “daily frequency of meals” was selected and used to calculate the RFS score. Participants were assigned one point for each recommended food and regular eating pattern (three meals a day) if they ate it at least once a week or more. The food items (and their corresponding points) for the RFS were as follows; daily frequency of meals (1), grains (1), legumes (4), vegetables (16), seaweeds (2), fruits/juices (12), fish (5), dairy products (3), nuts (1), and tea (1). Then, the score ranged from 0 to 46 points, and a higher score implies a better diet quality. The food items and their corresponding points for the RFS are shown in **Supplementary Table 2**. Subjects with increased consumption of foods rich in high antioxidant nutrients were given a higher score and lower scores to issues with lower consumption. All these antioxidant foods are healthy, and bad/unhealthy foods like sugar or sweets were not considered in the construction of this RFS.

### 2.7 HisCoM-RFS based on the HisCoM model

**Figure 1:**
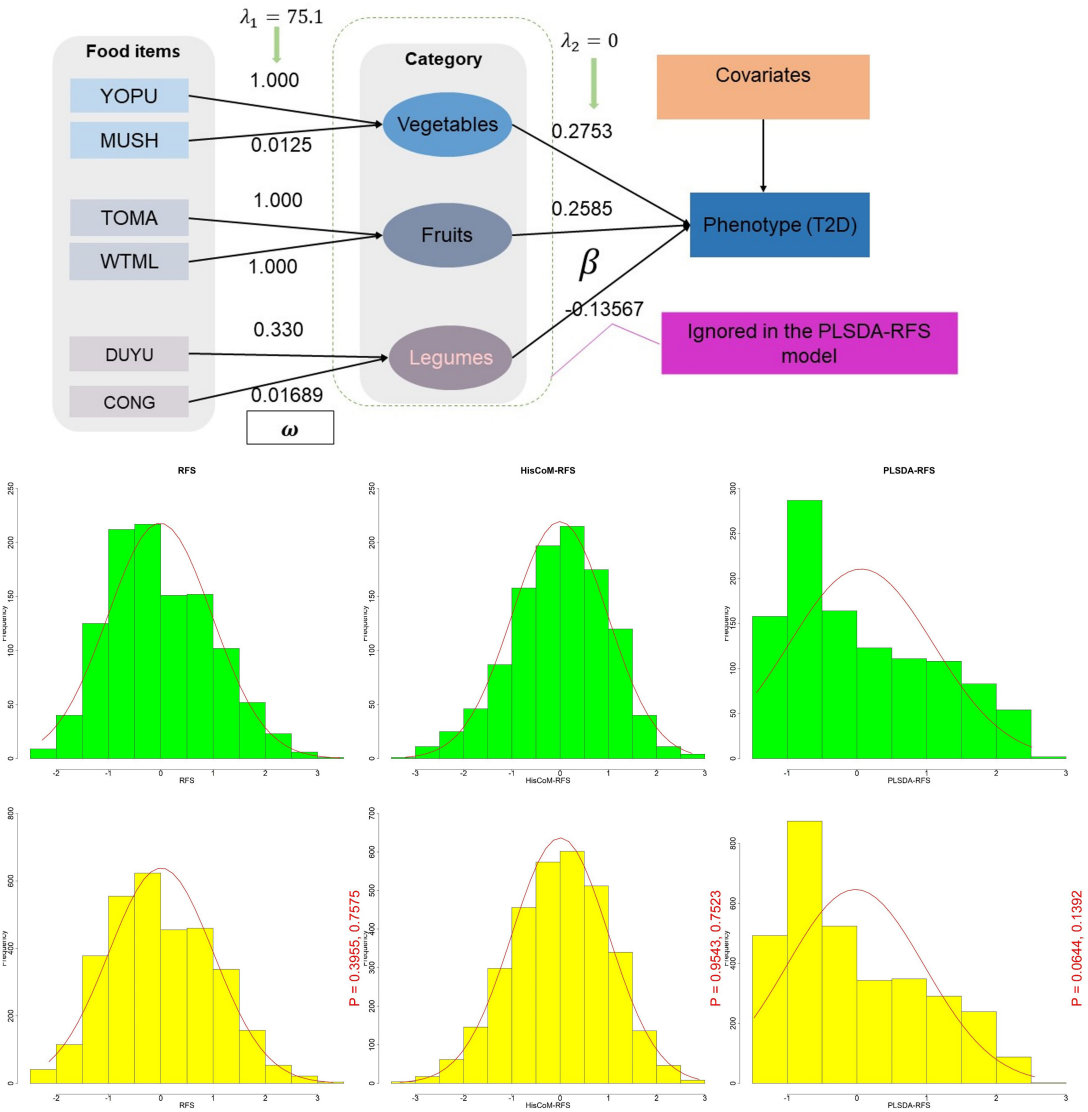
**(A)** A schematic diagram of the HisCoM model showing HisCoM-RFS calculation using three food categories. The rectangles and ellipses represent food items and food categories, respectively. So that the phenotype is a linear combination of the food items and not the food categories. HisCoM, Hierarchical Structural Component model. **(B)** Density plot distribution of RFS, HisCoM-RFS, and PLSDA-RFS between T2D (green) and control subjects (yellow). The p-values are from the Wilcoxon rank sum test and the Kolmogorov-Smirnov test, respectively between green and yellow. RFS, Recommended Food Scores; HisCoM, Hierarchical Structural Component model; PLS-DA, Partial Least Squares-D-iscriminant Analysis method; HisCoM-RFS, weighted RFS calculated by HisCoM model; PLSDA-RFS, weighted RFS calculated by PLS-DA method.

The calculated RFS assumes that each food item in a given category contributes equally to the diet quality of an individual. However, it is more reasonable to think that some food items contribute more than others. Therefore, we calculated a weighted food score using the RFS called HisCoM-RFS (Hierarchical Structural Component model (HisCoM) to analyze food scores) to capture this information. HisCoM estimates each food item’s weight and the significance level between the food category and the outcome T2D. The HisCoM used here (**Figure 1A**) is a modification of the Pathway-based approach using HierArchical components of collapsed RAre variants Of the High-throughput sequencing data (PHARAOH) model (26) that was developed by our laboratory. PHARAOH employs ridge penalization to control for any correlations between variables. It assumes that the biomarkers have a linear relationship with a phenotype of interest while analyzing entire pathways simultaneously.

For HisCoM, let $y_{i}$ define a phenotype (T2D) of the $i^{\text{th}}$ ( i=1,.,N)  subject and assume that it independently follows an exponential family distribution. Let $T_{k}$ be the number of food items in the $k^{\text{th}}$ food category. Let $g_{itk}$ denote the food score of the $t^{\text{th}}$ item in the $k^{\text{th}}$ food category for the $i^{\text{th}}$ subject. Let $w_{itk}$ denote a weight assigned to $g_{itk}$ and $\beta_{k}$ indicate the coefficient connecting the $k^{\text{th}}$ food category to the phenotype. Specifically, the relationship between the food scores of each food item and the case–control phenotype is established in such a way that;


$$logit\left(\pi_{i}\right)=\beta_{0}+\;\sum_{k=1}^{K}[\sum_{t=1}^{T_{k}}g_{itk}w_{itk}]\beta_{k}$$

Therefore, HisCoM-RFS is calculated as follows:


$$\text{HisCoM-RFS }=\sum_{k=1}^{K}[\sum_{t=1}^{T_{k}}g_{itk}w_{itk}]\beta_{k}$$

where  HisCoM estimated w and β . The $logit\left(\pi_{i}\right)$ is the logit function from logistic regression models explaining the log of odds (ratio of T2D subjects to normal subjects).

To estimate parameters HisCoM, the alternating least squares (ALS) algorithm was used, which was initially proposed by de Leeuw et al. (27) and adopted for the generalized structured component analysis (GSCA) (28) and later for the penalized log-likelihood function (26). PHARAOH employed the ALS algorithm in its penalized log-likelihood part (26). This ALS algorithm consists of two steps that iterate until convergence;

*Step 1*: For fixing the weight coefficient estimates $w_{itk}$ , update the food category coefficient estimates $\beta_{k}$ , in the sense of least squares.

*Step 2*: For fixing food category coefficient estimates $\beta_{k}$ , update the weight coefficient estimates $w_{itk}$ , in the sense of least squares.

We use a penalization approach to consider potential correlations between food items and categories. In this study, we adopt a ridge-type penalty to control multicollinearity between food items ( ${\text{λ}}_{1})\;$ only and not between food categories $\left({\text{λ}}_{2}=0\right)$ so that the phenotype is a linear combination of the food items and not the food categories. The significance of the estimated parameters was tested through the permutation by resampling the phenotypes.

### 2.8 PLSDA-RFS based on the PLS-DA method

In addition to the HisCoM method, another weighted food score called PLSDA-RFS was calculated using the commonly known partial least squares regression (PLS-R) for discriminant analysis (PLS-DA) method (29). PLS-DA is derived from PLS-R, where the response vector assumes discrete values (T2D) and considers the correlation between T2D and the food items while maximizing the covariance between T2D and the weights calculated (30, 31). PLS-DA incorporates T2D and RFS information in defining the scores and loadings (weights) used to calculate PLSDA-RFS. However, PLS-DA does not consider the food groups during the weight and coefficient calculation. PLSDA-RFS was calculated by multiplying the previously calculated unweighted RFS scores with the estimated weight matrix and the coefficient values in the first column of the estimated coefficient matrix.

### 2.9 Statistical analyses

Unless specified, statistical analyses were conducted using R software (version 4.2.1) to identify the association between T2D and diet. Categorical and continuous variables for participants’ general characteristics according to the case–control study for T2D were compared using the chi-squared test ( $\chi^{2}$ test) and two-sample t-test, respectively. The generalized linear regression model (GLM) was used to find the association ORs (95% CI) between diet (RFS, HisCoM-RFS, and PLSDA-RFS) and T2D. Secondly, the food scores were grouped into low, intermediate, and high diet quality groups and their ORs (95% CI) were estimated. After ranking the food scores, all food scores with ranks below 33.33% were grouped as low, intermediate for those below 66.6%, and above 66.6% as high. Thirdly, since genetic and lifestyle factors influence the development of T2D, gene–diet interaction analysis focused on the “interaction effect,” unlike the “main effect” between SNPs and food scores, was performed to identify SNPs, genes, and pathways associated with T2D. A significant interaction shows the role of dietary habits affecting pathways during the development of T2D. Logistic regression in PLINK (v1.90, Windows) was used for this analysis (19, 32). The analyses were adjusted for age, sex, area, BMI, smoking, alcohol consumption, coffee consumption, education level, income level, and METs following other studies involving KoGES Ansan–Ansung data (2, 16, 33–37). A statistical significance level of *P*< 0.05 was used unless specified. To find genes and pathways, significant SNPs from the interaction effect were mapped to genes and then pathways using the Multi-marker Analysis of GenoMic Annotation (MAGMA, windows version) tool, a generalized gene-set analysis tool of GWAS data (38, 39). MAGMA analyzes genes and pathways by multiple linear regression after principal component analysis for each gene. Pathway information was obtained from the Kyoto Encyclopedia of Genes and Genomes (KEGG) (40) database, whereas the gene location file (GRCh37) was downloaded from the National Center for Biotechnology Information (NCBI) website. Lastly, a global polygenic risk score (PRS) for T2D was generated using independent summary statistics (N = 191,764; 36,614 cases and 155,150 controls) from Biobank Japan (**Supplementary Figure 1**) (41). LDpred (42) was used to reweight each variant according to (1) the effect size, (2) the strength of statistical significance observed for T2D, and (3) linkage disequilibrium (LD) between a variant and others nearby. A tuning parameter that denotes the proportion of causal variants (P) estimated with the validation samples (P = 0.1) was selected. Nine categories capturing the interactions between genetic risk (low (reference), intermediate, and high) based on PRS and diet quality (low, intermediate, and high (reference)) based on the food scores were created. Adjusted ORs of the nine categories were calculated.

## 3 Results

### 3.1 Baseline characteristics of the subjects

A total of 350,000 SNPs and 8,840 subjects were left after genotype data QC. Diagnosis of the subjects for T2D left us with 4,975 subjects (1,288 cases and 3,687 control). The control subjects here are normal subjects without T2D. After adjusting for the covariates (for age, sex, area, BMI, smoking, alcohol consumption, coffee consumption, education levels, income levels, and METs), 4,292 (1,090 cases and 3,202 control) subjects were left. The characteristics of the 4,292 subjects are summarized in **Table 1**, presented as means ± standard deviation for continuous variables and percentage proportions for categorical variables. Smoking and alcohol consumption were left with two groups after data preprocessing. Income was summarized into five levels (<0.5~1, 1.0~2.0, 2.0~3.0, 3.0~4.0, >4.0), education into four classes (combined college, university, and graduate school into one level of higher education) and coffee consumption into four groups (never/seldom, monthly, weekly, and daily) in all analyses, to reduce on the number of levels of these variables. T2D was significantly associated with the area, sex, age, BMI, smoking, education level, income level, coffee consumption, PLSDA-RFS, and PRS.

**Table 1:** Baseline characteristics and food scores of participants according to T2D case–control subjects.

T2D case–control study			
	Case (n = 1,090)	Control (n = 3,202)	*P-value*
*Area*			3.6E-07
Ansung	511 (46.88)	1,219 (38.07)	
Ansan	579 (53.12)	1,983 (61.93)	
*Sex*			3.8E-06
Male	589 (54.04)	1,469 (45.88)	
Female	501 (45.96)	1,733 (54.12)	
*Age*	55.77 ( ± 8.76)	49.65 (8.26)	< 2.2E-16
*BMI (kg/m^2^)*	25.59 (± 3.27)	24.13 (± 2.89)	< 2.2E-16
*METs*	42.92 (± 24.84)	42.00 (± 23.97)	0.284
*Alcohol consumption*			0.077
Non-drinkers	590 (54.13)	1,632 (50.97)	
Former drinkers	500 (45.87)	1,570 (49.03)	
Current drinkers	0 (0)	0 (0)	
*Smoking*			0.033
Never (non-smoker)	0 (0)	0 (0)	
Former smoker	829 (76.06)	253 (79.20)	
Occasional smoker	261 (23.94)	666 (20. 80)	
Habitual smoker	0 (0)	0 (0)	
*Education level*			< 2.2E-16
≤ Elementary school	444 (40.73)	817 (25.52)	
Middle school	221 (20.28)	726 (22.67)	
High school	286 (26.24)	1,159 (36. 20)	
College	28 (2.57)	139 (4.34)	
University	99 (9.08)	301 (9.40)	
Graduate school (higher)	12 (1.10)	60 (1.87)	
*Income level (million Won)*			< 2.2E−16
<0.5	271 (24.86)	426 (13.30)	
0.5~1	172 (15.78)	428 (13.37)	
1.0~1.5	158 (14.50)	511 (15.96)	
1.5~2.0	142 (13.03)	465 (14. 52)	
2.0~3.0	160 (14.68)	690 (21.55)	
3.0~4.0	96 (8.81)	407 (12.71)	
4.0~6.0	61 (5.60)	199 (6.21)	
>6.0	30 (2.75)	76 (2.37)	
*Coffee consumption frequency*			0.0006
Never or seldom	299 (27.43)	686 (21.42)	
Once a month	35 (3.21)	99 (3.09)	
1–2 weeks	36 (3.30)	83 (2.59)	
2–3 weeks	79 (7.25)	232 (7.25)	
3–4 weeks	65 (5.96)	228 (7.12)	
5–6 weeks	25 (2.29)	104 (3.25)	
One daily	321 (29.45)	926 (28.92)	
Twice daily	108 (9.91)	406 (12.68)	
>3 daily	122 (11.19)	438 (13.68)	
Recommended Food Scores			
RFS	17.02 (7.53)	17.18 (7.56)	0.545
HisCoM-RFS	−0.16 (0.23)	−0.16 (0.23)	0.828
PLSDA-RFS	0.02 (0.01)	0.02 (0.01)	0.023
PRS	−1.54 (0.20)	−1.65 (0.20)	<2.2E−16

N = 4,292; values are n (%) for categorical variables and mean ± SD for continuous variables. Differences in characteristics were analyzed using χ^2^ tests for categorical variables and two-sample t-tests for continuous variables. METs, metabolic equivalents; RFS, Recommended Food Scores; PRS, polygenic risk scores.

### 3.2 HisCoM-RFS and PLSDA-RFS

The HisCoM and PLS-DA methods estimated the weights and coefficients ( β) used to calculate the weighted food scores HisCoM-RFS and PLSDA-RFS, as shown in **Figure 1A**. HisCoM estimated the weights of the 45 food items, the daily frequency of meals, and the coefficients of the 10 food categories and is shown in **Supplementary Tables 2**, **3**. PLS-DA also estimated the coefficients and weights of the 45 food items and the daily frequency of meals and was used to calculate PLSDA-RFS. The density plots of RFS, HisCoM-RFS, and PLSDA-RFS for control subjects (yellow; n = 3,347; bandwidth = 1.351, 0.04067 and 0.002164, respectively) and T2D subjects (green, n = 1,159; bandwidth = 1.653, 0.04929 and 0.002787, respectively) are shown in **Figure 1B**. There are no noticeable differences between the density plots between T2D and control subjects but between the food scores. We compared the distribution of the food scores between case and control subjects using the Wilcoxon rank-sum test and the Kolmogorov–Smirnov test. The P-values of the tests are shown in **Figure 1B**. The Wilcoxon rank-sum test showed that the two groups are not different, whereas the Kolmogorov–Smirnov test showed that the two groups come from the same distribution.

### 3.3 Association between diet quality measured using food scores and T2D

**Figure 2:**
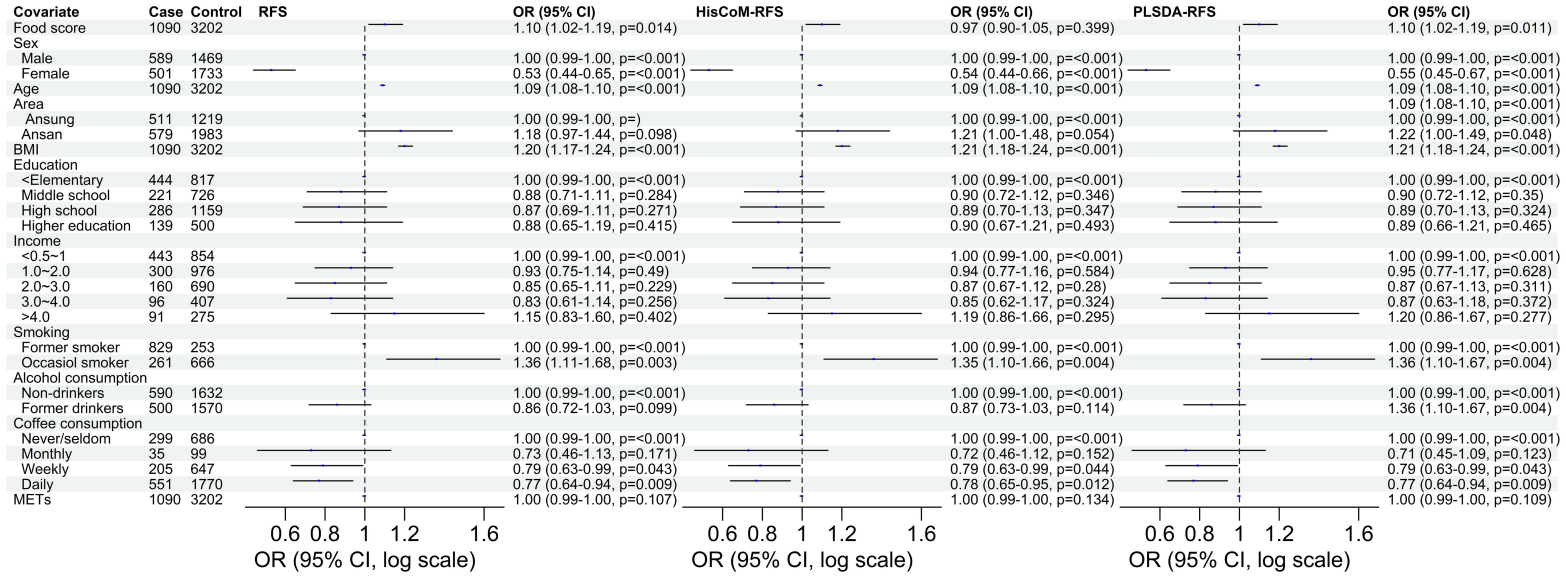
The odd ratios of RFS, HisCoM-RFS and PLSDA-RFS adjusted for age, sex, area, BMI, income level, education level, smoking, alcohol and coffee consumption, and METs, showing the association between dietary habits and T2D. The *P*-values were calculated using multiple logistic regression. METs, metabolic equivalents; BMI, body mass index; RFS, recommended food scores.

**Figure 3:**
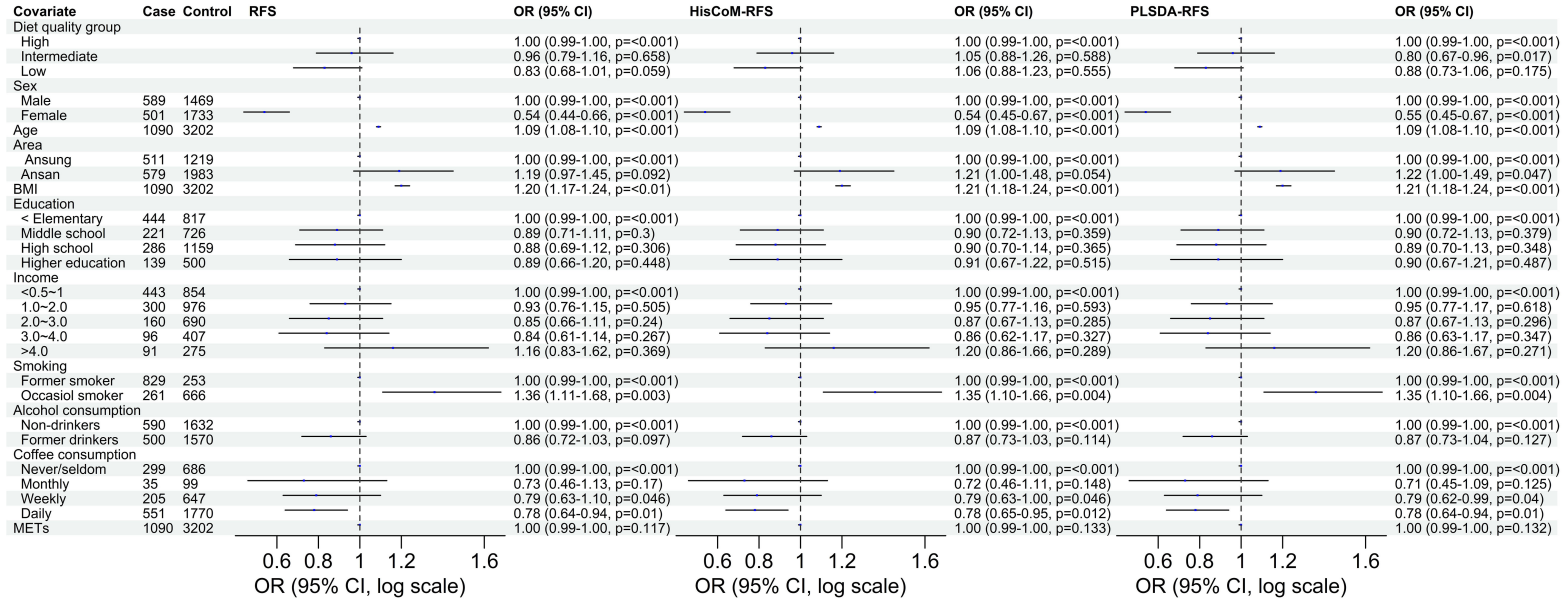
The odd ratios of RFS, HisCoM-RFS and PLSDA-RFS diet quality groups adjusted for age, sex, area, BMI, income level, education level, smoking, alcohol and coffee consumption, and METs, showing the association between dietary quality and T2D. High diet quality group is the reference group. The *P*-values were calculated using multiple logistic regression. METs, metabolic equivalents; BMI, body mass index; RFS, recommended food scores.

Of the three food scores, only PLS-DA was positively associated with T2D unadjusted for the other covariates (OR: 1.0839; 95% CI: 0.9293–1.0622; *P* = 0.0203). The food scores were standardized for comparable results. After adjusting for potential covariates (age, sex, BMI, and area) and lifestyle factors (smoking, alcohol and coffee consumption, education level, income level, and METs), both RFS (OR: 1.11; 95% CI: 1.03–1.20; *P* = 0.014) and PLSDA-RFS (OR: 1.10; 95% CI: 1.02–1.19, *P* = 0.011) were positively associated with T2D, as shown in **Figure 2**. This indicates that a person’s dietary patterns can affect the development of T2D. Grouping the food scores into low, intermediate, and high diet quality groups, with high being the reference group, showed the low diet quality group of RFS (OR: 0.83; 95% CI: 0.68–1.01, *P* = 0.059) and the intermediate diet quality group of PLSDA-RFS (OR: 0.80, 95% CI: 0.73–1.06; *P* = 0.017) to be associated with T2D, as shown in **Figure 3**. From the two analyses, being female, age, BMI, being an occasional smoker, and at least weekly and daily consumption of coffee were constantly associated with T2D (*P*< 0.05).

### 3.4 Gene–diet interaction analysis

**Figure 4:**
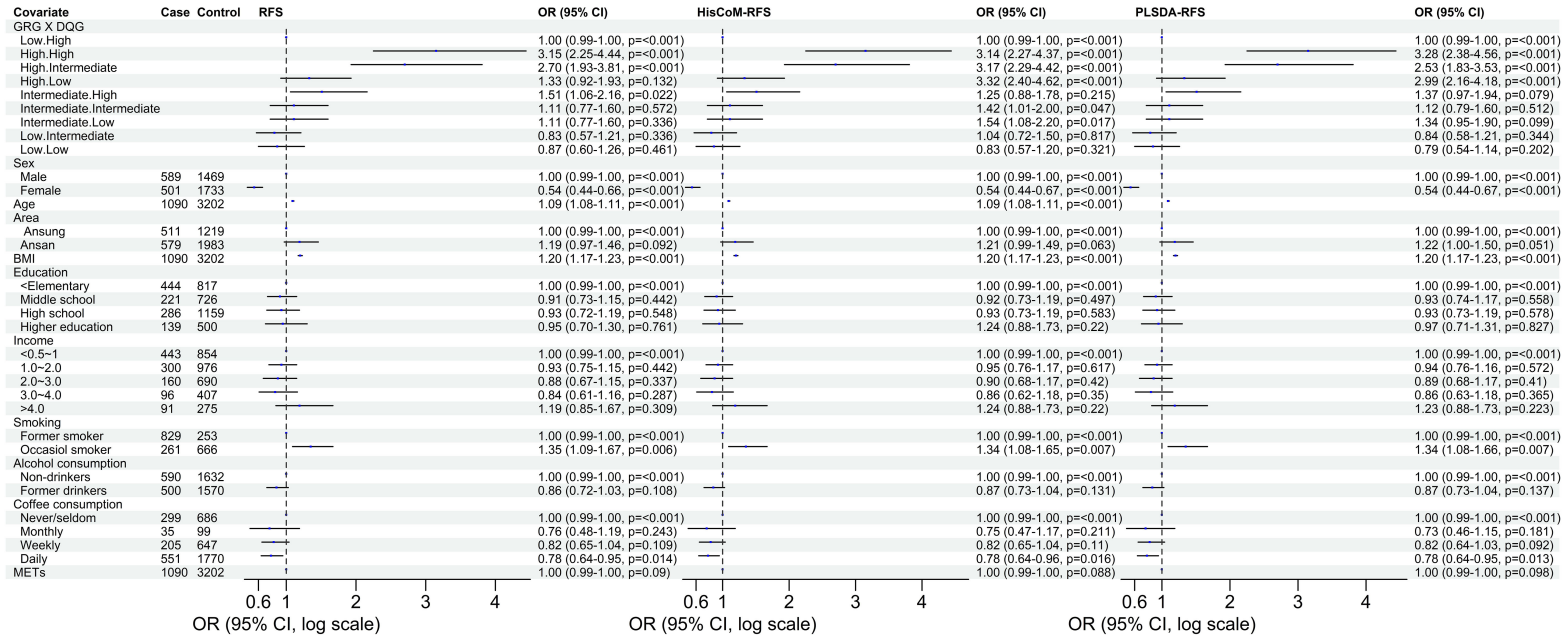
The odd ratios of the interaction between the genetic risk groups and diet quality groups of RFS, HisCoM-RFS and PLSDA-RFS. The analyses were adjusted for the ten covariates. Interaction of low GRC and high DQG is the reference group. METs, metabolic equivalents; BMI, body mass index; RFS, recommended food scores; GRG, genetic risk groups; DQG, diet quality groups.

The 8,205, 9,331, and 103,408 SNPs for RFS, HisCoM-RFS, and PLSDA-RFS, respectively (3,301, 59, and 4,260 SNPs were significant at *P*< 0.001), were significant with “interaction effect” *P*< 0.05. Their Manhattan plots are shown in **Supplementary Figure 1**. These SNPs were mapped to genes and pathways using the bioinformatics tool MAGMA. **Table 2** shows the 19 and 29 genes (12 common genes) found in the gene analysis step of MAGMA using RFS and PLSDA-RFS, respectively, at *P*< 1.0E-08. HisCoM-RFS did not yield any significant genes at *P*< 1.0E-08. The 12 genes common to both RFS and PLSDA-RFS include FHIT, CACNA2D3, ITPR1, RELN, CNTNAP2, CTNNA2, DOCK2, ROBO2, SLIT3, MAG12, ASIC2, and CREB5. **Supplementary Table 4** shows the pathways from the pathway analysis (or gene-set analysis) step of MAGMA for RFS, HisCoM-RFS, and PLSDA-RFS at *P*< 0.05. Pathway analysis found some of the pathways to be associated with T2D in literature, for example, pathways such as the insulin signaling pathway, adipocytokine signaling pathway, type II diabetes, prostate cancer, and other metabolic pathways (43–49). Multiple comparison corrections of the discovered pathways using the false discovery rate (FDR) correction method found vascular smooth muscle contraction (q-value = 0.06), small cell lung cancer (q-value = 0.007), long-term potentiation (q-value = 0.065), and adipocytokine signaling (q-value = 0.026) pathways (FDR q-value< 0.1). The strongest association was observed with the adipocytokine signaling pathway yielding a significant gene set of 32 genes listed in **Table 3**. Some genes include STAT3, AKT1-AKT3, MAPK10, MAPK8, PRKAA1, ACSL1, CAMKK1 RXRG, and NFKB. Finally, genetic risk assessed using global PRS showed a strong positive association with T2D adjusted for the covariates (OR: 17.78; 95% CI: 12.01–26.50; *P*< 0.01). Grouping of the PRS into low, intermediate, and high genetic risk groups with the low genetic risk as the reference as shown in **Supplementary Figure 3** showed both intermediate (OR: 1.46; 95% CI: 1.19–1.79; *P*< 0.01) and high (OR: 3.36; 95% CI: 2.77–4.08; *P*< 0.01) genetic risk groups having an association with T2D, as shown in **Supplementary Figure 3**. The nine groups showing interactions between diet quality groups and genetic risk groups found significant interactions between different genetic risk groups and diet quality groups, as shown in **Figure 4**, especially the high GRC and the diet quality groups.

**Table 2:** List of significant genes from the gene analysis step of MAGMA for RFS and PLSDA-RFS.

RFS				PLSDA-RFS			
GENE	CHROMOSOME	No. of SNPs	*P*	GENE	CHROMOSOME	No. of SNPs	P
ASIC2	17	78	6.48E−14	ASIC2	17	111	1.13E−13
CACNA2D3	3	59	6.58E−09	CACNA1C	12	34	3.17E−11
CREB5	7	97	1.07E−10	CACNA2D3	3	75	1.40E−11
CTNNA2	7	48	3.01E−11	CACNB3	10	49	2.44E−09
DOCK2	2	56	2.38E−10	CDH4	20	43	1.73E−09
ERBB4	5	45	1.07E−10	CNTNAP2	7	124	1.89E−11
FGF12	2	34	3.29E−10	CREB5	7	39	6.5E−10
FHIT	3	32	3.56E−09	CTNNA2	2	50	1.56E−11
GABRG3	3	94	1.51E−11	CTNNA3	10	92	2.95E−12
GRM7	15	17	4.13E−09	DOCK2	5	30	1.36E−09
ITPR1	3	67	3.75E−10	FGF14	13	45	7.60E−11
KCNMA1	3	27	1.29E−09	FHIT	3	92	6.51E−11
MAGI2	10	45	2.88E−10	FMN2	1	34	3.05E−09
MAGI2	7	83	1.28E−10	GALNT18	11	26	2.86E−09
PDE4D	5	50	8.41E−09	ITRP1	3	26	8.30E−09
RELN	7	39	1.06E−09	MAGI2	7	67	6.97E−12
ROBO2	3	95	8.53E−12	NRXN1	2	66	5.39E−09
RYR3	15	28	2.05E−09	NRXN3	14	64	1.47E−09
SLIT3	3	58	3.62E−09	PRKCA	17	33	2.04E−09
				PRKCE	2	31	3.28E−11
				PRKG1	10	65	5.20E−10
				PSD3	8	42	2.35E−09
				PTPRN2	7	35	7.74E−09
				RELN	7	38	7.72E−09
				ROBO2	3	71	2.13E−11
				RPS6KA2	6	46	5.93E−11
				RYR2	1	41	9.98E−11
				SLIT3	5	55	5.43E−11
				UST	6	19	1.51E−09

**Table 3:** List of genes from the gene set of the adipocytokine signaling pathway.

Gene	Chromosome	No. of SNPs	*P*
ACACB	12	9	0.0003
ACSL1	4	4	0.0058
ACSL3	2	5	0.0089
ACSL5	10	5	0.0096
ACSL6	5	5	0.007
ADIPOR2	12	2	0.0042
AKT1	14	2	0.0144
AKT2	19	2	0.015
AKT3	1	4	0.0135
CAMKK1	17	1	0.0002
CAMKK2	12	2	0.0106
CD36	7	3	0.0004
IKBKB	8	1	0.0014
IRS1	2	6	0.0031
LEPR	1	2	0.0167
MAPK10	4	9	0.0033
MAPK8	10	9	0.0014
NFKB1	4	14	0.0022
NPY	7	1	0.0007
POMC	2	1	0.04
PPARA	22	1	0.012
PPARGC1A	4	35	1.32E-07
PRKAA1	5	2	0.0332
PRKAA2	1	2	0.0047
PRKAG2	7	4	0.0006
PRKCQ	10	18	3.7E−05
RXRA	9	1	0.003
RXRG	1	2	0.0071
SLC2A1	1	1	0.0059
STAT3	17	4	0.0413
TNFRSF1A	12	1	0.0033

## 4 Discussion

Investigating the role of dietary patterns in association with T2D is still a hot research topic worldwide. A previous study showed that a higher RFS score is associated with lower oxidative stress but failed to show an association with T2D in Korean adults (16). To maximize the interaction power of RFS on T2D, we developed weighted RFS scores HisCoM-RFS and PLSDA-RFS using the HisCoM and PLS-DA models, which determine the weights of the food items. The development of weighted food scores is based on the assumption that some food items contribute more than others to the overall food score given the phenotype. After calculating the weighted food scores, we performed analyses contrasting these food scores about the association of dietary patterns with the development of T2D while adjusting for covariates and other lifestyle factors like METs, smoking, alcohol and coffee consumption, and education and income levels. Firstly, a significant association was found between dietary habits, mainly with the weighted food score PLSDA-RFS, unlike the former unweighted RFS score in the previous study (16). After adjusting the 10 covariates, unweighted RFS and PLSDA-RFS food scores were significantly associated with T2D. Grouping the food scores into low, intermediate, and high diet quality groups showed intermediate and low diet quality groups to be associated with T2D. This shows the importance of high diet quality (foods rich in antioxidant properties) playing a preventive role in the occurrence of T2D.

In the literature, a higher Dietary Approaches to Stop Hypertension (DASH) Score was associated with lower T2D risk in men (50). An extended follow-up of urban Chinese adults showed that a higher healthy diet score (HDS) was associated with lower diabetes risk (51). Other studies also associated diet quality with the risk of T2D (9, 52). Secondly, interaction analysis between food scores and SNPs focusing on the “interaction effect” instead of the “main effect” aimed to find genes and pathways associated with T2D. Significant interaction implies that diet is involved with pathway mechanisms related to the development of T2D. The interaction analysis with the respective food scores RFS, HisCoM-RFS, and PLSDA-RFS yielded some significant SNPs (*P*< 0.05), filtered and used in MAGMA’s gene and pathway analysis steps. We did not get any SNPs below the GWAS significance level of *P*< 5.0E-08. Gene analysis yielded 19 genes and 29 genes at *P*< 1.0E-08 with RFS and PLSDA-RFS, respectively, with 12 common genes, namely, FHIT, CACNA2D3, ITPR1, RELN, CNTNAP2, CTNNA2, DOCK2, ROBO2, SLIT3, MAGI2, ASIC2, and CREB5. FHIT is involved in purine metabolism, and CACNA2D3 is engaged with the voltage-dependent calcium channel. Calcium signaling is crucial for insulin secretion in pancreatic cells (53, 54). RELN gene encodes a large secreted extracellular matrix protein thought to control cell–cell interactions critical for cell positioning and neuronal migration during brain development and is involved in multiple disorders. CTNNA2 enables actin filament binding activity, whereas DOCK2 remodels the actin cytoskeleton required for lymphocyte migration in response to chemokine signaling. SLIT3 is associated with cell receptors during cellular migration (55).

The pathway analysis revealed many pathways, some of which have been associated with T2D in literature. The pathways are mainly related to cancer, metabolism, and signaling. However, FDR correction left vascular smooth muscle contraction (*q*-value = 0.06), small cell lung cancer (*q*-value = 0.007), long-term potentiation (*q*-value = 0.065), and adipocytokine signaling (*q*-value = 0.026) pathways to be strongly associated with T2D at q-value< 0.1. The strongest association was observed with the adipocytokine signaling pathway, which produced a gene set of 32 genes in this pathway strongly associated with T2D. These genes include STAT3, AKT1-AKT3, MAPK10, MAPK8, IRS1, ADIPOR2, ACSL1, CAMKK1, RXRG, and NFKB1. STAT3 is involved in cytokine- and nutrient-induced insulin resistance, and its phosphorylation contributes to skeletal muscle insulin resistance in T2D (56). MAPK10 was identified as a critical gene in diabetes mellitus-induced atrial fibrillation in mice (57). The AKT genes and IRS1 may influence adipocyte insulin resistance (58–61). Variants in the ADIPOR2 gene are associated with increased diabetic risk (62, 63). In a meta-analysis study, RXRG, NFKB1, ACSL1, and CAMKK1 genes were also associated with T2D (64). Briefly, insulin resistance is one of the major hallmarks of the pathogenesis and etiology of T2D (48). It is reflected by impairments in insulin signaling in the diabetic state displaying a reduced insulin sensitivity (43). A generally accepted view is that insulin resistance associated with T2D is caused by defects at one or several levels of the insulin-signaling cascade, for example, in skeletal muscles, adipose tissue, and liver, that quantitatively constitute the bulk of the insulin-responsive tissues (45). Adipocytes and resident macrophages that have migrated to the adipose tissue produce and secrete adipocytokines, including tumor necrosis factor-α, interleukin-6, resistin, and adiponectin, which are thought to contribute to the development of insulin resistance and T2D (46, 47, 64). Dysregulation of vascular smooth muscle excitability using calcium ions occurs during T2D disorder (65–67). Abnormal long-term potentiation behavior is observed in patients with T2D (68, 69).

One limitation of the study is that the genotype data were not imputed with the 1000G population data when the analysis was carried out. Also, larger cohort data are needed to replicate these findings. In the future, we will perform the same analysis using genotype data imputed using 1000G and replicate the findings of our analysis using an independent dataset.

In conclusion, this study revealed the association between dietary patterns and the development of T2D. The risk of T2D increases in individuals with poor dietary habits (foods lacking antioxidant properties). Lifestyle habits like smoking, BMI, age, and alcohol and coffee consumption increase the risk of T2D. The impact of genetics was also observed, especially in people with high genetic risks. The interaction between diet and genetics showed that dietary patterns affect pathway mechanisms in the development of T2D. The study results elucidate the protective role of a healthy diet in lowering the risk of T2D. However, further prospective investigations, more rigorous studies of larger cohorts, intervention research, or different methods of constructing food (indices) quality scores will be needed to investigate if diet can predict the prevalence of T2D (causal–effect relationship). Also, further validation studies of the above pathways are required to find T2D biochemical pathogenesis conclusively.

## Data availability statement

The data analyzed in this study is subject to license/restrictions: Data supporting this paper were obtained from the National Biobank of Korea (NBK). The KoGES epidemiology data and the KARE genotype data are available only upon agreement with NBK. Requests to access these data can be directed to National Human Resources Bank (http://biobank.nih.go.kr 1661-9070) or the Human Biobank Information System (HuBIS desk) at https://is.kdca.go.kr/. BBJ summary statistics used in this study were downloaded from the Biobank Japan PheWeb: https://pheweb.jp/.

## Author contributions

The authors’ responsibilities were as follows: conceptualization: TP. Methodology: TP and CA. Formal analysis: CA. Resources: TP and OK. Writing—original draft preparation: CA. Writing—review and editing: CA, TP, WC, and MM. Supervision: TP. Project administration: TP. Funding acquisition: TP. All authors contributed to the article and approved the submitted version.
