# Supplementary material for: Gene–diet interaction analysis using novel weighted food scores discovers the adipocytokine signaling pathway associated with the development of type 2 diabetes
Source: Front Endocrinol (Lausanne). 2023 Aug 23;14:1165744. doi: 10.3389/fendo.2023.1165744 (PMC10482093; doi:10.3389/fendo.2023.1165744)
Supplement: Supplementary file 1 [file DataSheet_1.pdf]

Supplementary Material

Gene-diet interaction analysis discovers mainly adipocytokine signaling pathway associated with the pathogenesis of Type 2 Diabetes

Catherine Apio[1], Wonil Chung[2], Min Kyong Moon[3], Oran Kwon[4]*, Taesung Park[5*]

*** Correspondence:** Professor Oran Kwon, E-mail: orank@ewha.ac.kr; Professor Taesung Park, Fax: 82-2-883-6144, E-mail: tspark@stats.snu.ac.kr

Supplementary Table 1. List of covariates

**Covariates**
Age
Sex
Area
BMI
Metabolic equivalents (METs)
Alcohol consumption
Smoking
Income
Education
Coffee consumption

Supplementary Table 2. Table showing the food items, good groups, and the weights of the food items used in the calculation of HisCoM-RFS estimated by the HisCoM model

Food Category	Food items	weights	*P*	Q-value
Dairy products	Cheese	0.1135	0.455	0.6663
Dairy products	Milk	0.2578	0.091	0.2203
Dairy products	Yogurt	1.0000	0.311	0.5722
Fish	Anchovy	0.2573	0.042	0.1932
Fish	Black blue Fish	0.2244	0.084	0.2198
Fish	BlackFish	0.1259	0.048	0.2007
Fish	Jogi	0.0385	0.478	0.6663
Fish	Pollack, dongtae	-0.5871	0.002	0.0184
Fruits	Apples or juice	0.5926	0.012	0.0863
Fruits	Banana or juice	0.4902	0.038	0.1932
Fruits	Grapes or juice	0.0556	0.596	0.7410
Fruits	Tangerine or juice	-0.1140	0.285	0.5463
Fruits	Melon	0.4405	0.001	0.0115
Fruits	Oranges or juice	0.1722	0.067	0.2116
Fruits	Pears	0.0564	0.567	0.7410
Fruits	Persimmon or dried persimmon	0.5302	0.085	0.2198
Fruits	Peaches or Plum	-0.1467	0.635	0.7572
Fruits	Strawberries	0.8014	0.001	0.0115

Fruits	Tomatoes	1.0000	0.001	0.0115
Fruits	Watermelons	1.0000	0.001	0.0115
Legumes	Soybeans	-0.0327	0.762	0.8763
Legumes	Doenjang soup or Cheonggukjank sooup	0.0790	0.46	0.6663
Legumes	Tofu	0.1299	0.233	0.466
Legumes	Soy milk	0.3640	0.015	0.0863
Meals	Meals	-0.1213	0.015	0.0863
Nuts	PeaNuts or almond Nuts or pine nut	0.0559	0.445	0.6663
Seaweed	Kelp or Seaweed	0.1363	0.642	0.7572
Seaweed	Seaweed	-0.0149	0.951	0.9630
Tea	Tea	-0.7316	0.581	0.7410
Vegetables	Leek or water parsley	0.1227	0.813	0.9121
Vegetables	Carrots	-0.3441	0.066	0.2116
Vegetables	Red pepper leaf	0.2701	0.181	0.3965
Vegetables	Perilla leaf	-0.0066	0.963	0.9630
Vegetables	Green pepper	0.3667	0.059	0.2116
Vegetables	Other Vegetables (watercress, mugwort, mustard, radish)	0.0103	0.963	0.9630
Vegetables	Cabbage or Chinese cabbage	0.1576	0.36	0.6369
Vegetables	Other mushrooms	-0.0135	0.932	0.9630

Vegetables	Oyster mushrooms	0.1091	0.472	0.6663
Vegetables	Onions	0.1325	0.408	0.6663
Vegetables	Cucumber	-0.2149	0.231	0.4660
Vegetables	Pumpkin or juice or pumpkin porridge	0.8693	0.069	0.2116
Vegetables	Lettuce	-0.0374	0.943	0.9630
Vegetables	Spinach	-0.6544	0.122	0.2806
Vegetables	Bean sprouts or sprouts	0.2756	0.559	0.7410
Vegetables	Zucchini	1	0.379	0.6457
Whole grain	Multigrain rice	0.2773	0.086	0.2198

Supplementary Table 3. The coefficients of the food groups are estimated from the HisCoM model used for the calculation of HisCoM-RFS.

**Food category**	**HisCoM**			
	Number of food items	β	*P value*	*Q value*
Fish	5	0.2646	0.0010	0.0030
Fruits	12	-0.2150	0.0010	0.0030
Vegetables	16	0.1320	0.3790	0.4740
Dairy products	3	0.0888	0.3110	0.4440
Legumes	4	-0.1290	0.0350	0.0875
Seaweed	2	-0.0293	0.7690	0.7690
Meals	1	-0.1644	0.0010	0.0033
Whole grain	1	-0.0378	0.2930	0.4443
Nuts	1	0.0494	0.1650	0.3300
Tea	1	-0.0134	0.7210	0.7690

Supplementary Table 4. List of pathways from the gene-set analysis (pathway analysis) step of MAGMA for RFS, HisCoM-RFS and PLSDA-RFS.

**Food score**	**SNP cut-off P-value**	**Pathway**	**# of genes**	**P-value**	**FDR**
RFS	< 0.001	TASTE TRANSDUCTION	2	0.015	0.534
		PHOSPHATIDYLINOSITOL SIGNALING SYSTEM	5	0.030	0.534
		HUNTINGTONS DISEASE	2	0.033	0.534
		GLYCEROPHOSPHOLIPID METABOLISM	5	0.035	0.534
		AMINO SUGAR AND NUCLEOTIDE SUGAR METABOLISM	2	0.046	0.534
		FRUCTOSE AND MANNOSE METABOLISM	2	0.046	0.534
		PROSTATE CANCER	2	0.048	0.534
	< 0.05	PENTOSE PHOSPHATE PATHWAY	10	0.002	0.458
		PROGESTERONE-MEDIATED OOCYTE MATURATION	31	0.013	0.964
		AMINOACYL TRNA BIOSYNTHESIS	12	0.017	0.964
		LYSINE DEGRADATION	14	0.021	0.964
		VALINE LEUCINE AND ISOLEUCINE BIOSYNTHESIS	4	0.029	0.964
		GLYCOLYSIS GLUCONEOGENESIS	16	0.046	0.964
HisCoM-RFS	< 0.05	**VASCULAR SMOOTH MUSCLE CONTRACTION**	**22**	**0.0005**	**0.066**

NON HOMOLOGOUS END JOINING	2	0.005	0.245
PPAR SIGNALING PATHWAY	2	0.006	0.245
INSULIN SIGNALING PATHWAY	12	0.010	0.245
B-CELL RECEPTOR SIGNALING PATHWAY	6	0.012	0.245
FC EPSILON RI SIGNALING PATHWAY	11	0.014	0.245
PATHWAYS IN CANCER	36	0.015	0.245
PHOSPHATIDYLINOSITOL SIGNALING SYSTEM	16	0.015	0.245
GNRH SIGNALING PATHWAY	18	0.016	0.245
ERBB SIGNALING PATHWAY	13	0.028	0.336
ALDOSTERONE-REGULATED SODIUM REABSORPTION	2	0.031	0.336
ADIPOCYTOKINE SIGNALING PATHWAY	3	0.033	0.336
CARDIAC MUSCLE CONTRACTION	6	0.033	0.336
TYPE II DIABETES MELLITUS	7	0.036	0.336
OLFACTORY TRANSDUCTION	8	0.040	0.336
PENTOSE PHOSPHATE PATHWAY	3	0.040	0.336
WNT SIGNALING PATHWAY	11	0.042	0.336
ALZHEIMERS DISEASE	11	0.043	0.336

PLSDA-RFS	< 0.001	**SMALL CELL LUNG CANCER**	**2**	**7.20E-05**	**0.007**
		**LONG TERM POTENTIATION**	**8**	**0.001**	**0.065**
		**VASCULAR SMOOTH MUSCLE CONTRACTION**	**16**	**0.002**	**0.069**
		GAP JUNCTION	12	0.009	0.217
		ALZHEIMERS DISEASE	9	0.011	0.225
		APOPTOSIS	3	0.022	0.242
		NON-SMALL CELL LUNG CANCER	2	0.024	0.242
		LONG TERM DEPRESSION	12	0.027	0.242
		ECM RECEPTOR INTERACTION	3	0.028	0.242
		TYPE II DIABETES MELLITUS	5	0.028	0.242
		NEUROTROPHIN SIGNALING PATHWAY	2	0.029	0.242
		DILATED CARDIOMYOPATHY	11	0.032	0.242
		P53 SIGNALING PATHWAY	2	0.037	0.242
		NATURAL KILLER CELL-MEDIATED CYTOTOXICITY	5	0.039	0.242
		HYPERTROPHIC CARDIOMYOPATHY HCM	8	0.040	0.242
		TOLL-LIKE RECEPTOR SIGNALING PATHWAY	3	0.042	0.242
		OOCYTE MEIOSIS	6	0.043	0.242

	FC GAMMA R MEDIATED PHAGOCYTOSIS	6	0.044	0.242
	FC EPSILON RI SIGNALING PATHWAY	3	0.046	0.242
< 0.05	**ADIPOCYTOKINE SIGNALING PATHWAY**	**32**	**0.0001**	**0.028**
	GLYCOSYLPHOSPHATIDYLINOSITOL GPI ANCHOR BIOSYNTHESIS	10	0.005	0.270
	PURINE METABOLISM	78	0.005	0.270
	SNARE INTERACTIONS IN VESICULAR TRANSPORT	14	0.006	0.270
	SPHINGOLIPID METABOLISM	17	0.010	0.364
	NOTCH SIGNALING PATHWAY	16	0.020	0.571
	LONG TERM DEPRESSION	39	0.025	0.571
	VASCULAR SMOOTH MUSCLE CONTRACTION	62	0.029	0.571
	DORSO VENTRAL AXIS FORMATION	18	0.035	0.571
	ARGININE AND PROLINE METABOLISM	21	0.040	0.571
	GLYCOSAMINOGLYCAN BIOSYNTHESIS CHONDROITIN SULFATE	10	0.043	0.571
	CARDIAC MUSCLE CONTRACTION	28	0.043	0.571
	TYPE II DIABETES MELLITUS	27	0.046	0.571

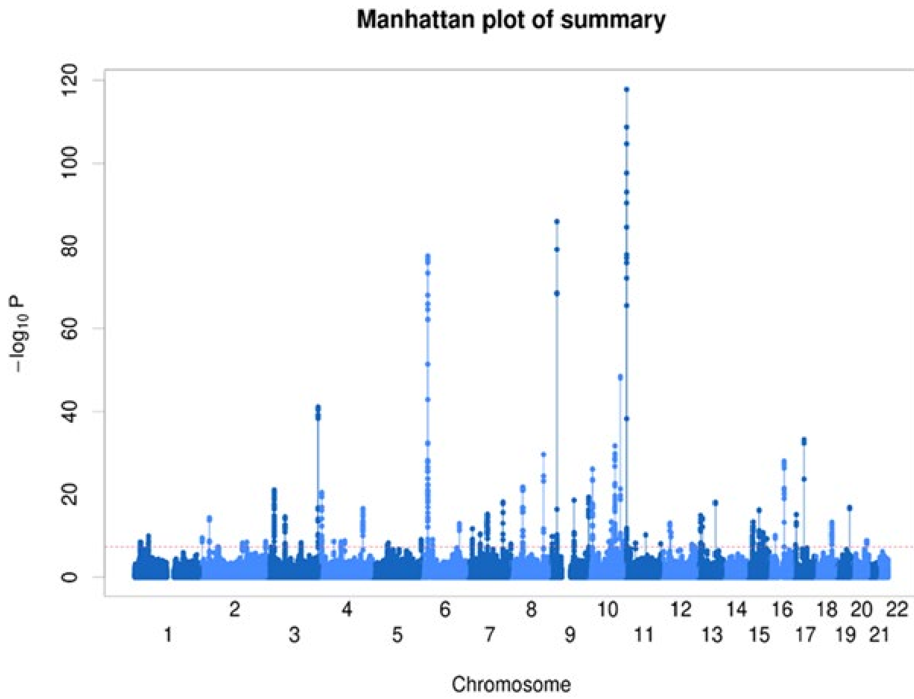

Supplementary Figure 1. Manhattan plot for T2D (N=191,764) using BBJ (Summary statistics are available at http://jenger.riken.jp/en/result).

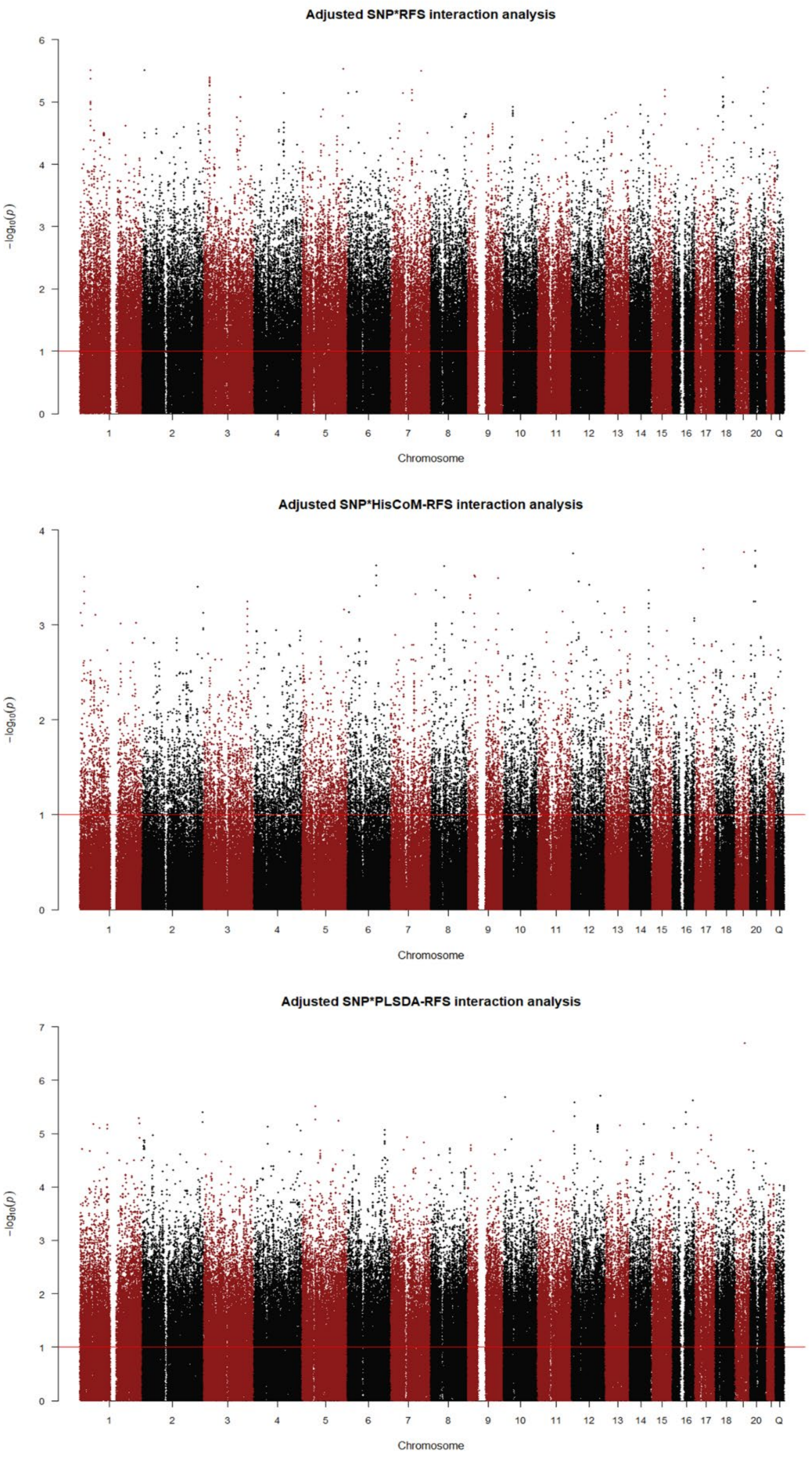

Supplementary Figure 2. Manhattan plot of the interaction between SNPs and food scores. Logistic regression in PLINK was used to calculate the P-values. The analysis was adjusted for age, sex, area, BMI, alcohol and coffee consumption, income and education levels, METs and smoking.

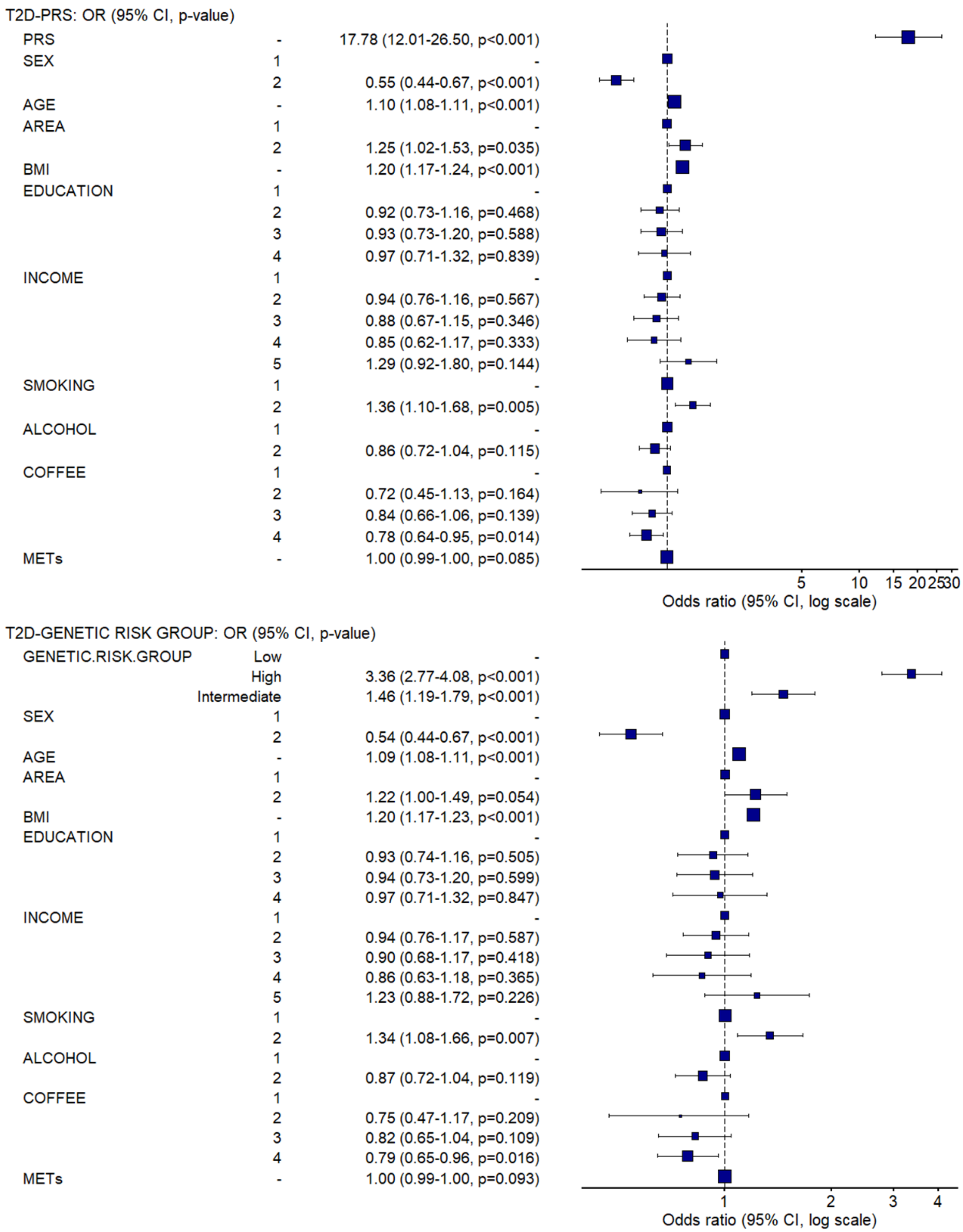

Supplementary Figure 3. Odd ratio plots of the association between T2D and the global polygenic risk scores (PRS) and the genetic risk groups (low, intermediate and high) adjusted for covariate.
